# Fecal shedding level of *Haemonchus contortus* is associated with gastrointestinal bacterial microbial composition in naturally infected sheep

**DOI:** 10.1590/S1984-29612025005

**Published:** 2025-02-03

**Authors:** Jeferson Vidart Ramos, Verônica Rocha Merched, Laura Dias da Silva Ribeiro, Guilherme Neves Lima Rattmann, Renan Eugênio Araújo Piraine, Fabio Pereira Leivas Leite

**Affiliations:** 1 Departamento de Microbiologia e Parasitologia, Universidade Federal de Pelotas – UFPel, Pelotas, RS, Brasil; 2 Departamento de Zootecnia, Universidade Federal de Pelotas – UFPel, Pelotas, RS, Brasil; 3 Centro de Desenvolvimento Tecnológico, Universidade Federal de Pelotas – UFPel, Pelotas, RS, Brasil

**Keywords:** Bacterial community profiling, helminth, host-parasite-bacteria, Perfil da comunidade bacteriana, helminto, hospedeiro-parasita-bactéria

## Abstract

A complex interaction of gastrointestinal parasites with sheep hosts may involve bacteria communities, parasite genera, parasitic genes, and biological pathways. *Haemonchus contortus* presents a global challenge for ruminants, and the bacterial community can influence sheep's resistance and susceptibility to these parasites. Thus, a better understanding of this complex interaction could contribute to the development of a new approach to parasite control. This study evaluated the bacterial community of Corriedale sheep naturally infected with *H. contortus* based on the fecal egg counts over ten months and then classified as having low (LC), intermediate (IC), or high (HC). Stool samples were collected monthly for egg counts (EPG), and 16S rRNA gene sequencing was performed on five animals from each group. The average EPG was 2,635 ± 105 for HC, 845 ± 129 for IC, and 110 ± 70 for LC, with a significant difference (p = 0.0001). Firmicutes, Proteobacteria, and Spirochaetes were more abundant in the HC group. 102 bacterial genera showed significant differences between the LC and HC groups. Beta diversity was statistically different (p<0.005) for HC compared with the other two groups; also, different communities were found between LC and HC. *Sediminispirochaeta, Oribacterium, Alloprevotella, Prevotellaceae*_UCG-001, *Prevotellaceae*_UCG-003, *Ruminiclostridium*_6 and *Ruminococcus*_1were significant more abundant in LC, and IC group. *Acetobacter* and *Methanocorpusculum* had a significant reduction in the LC group. Thus, bacterial genera related to low methane emission and food efficiency were significantly present in the LC group. Therefore, a better understanding of the role of host-bacterial community-parasite interaction could contribute to improving parasite control management.

## Resumo

A complexa interação entre parasitas gastrointestinais e hospedeiros ovinos envolve comunidades de bactérias, gêneros de parasitas, genes parasitários e vias biológicas. *Haemonchus contortus* representa um desafio global para ruminantes, e a comunidade bacteriana pode influenciar a resistência e a suscetibilidade a esses parasitas. Assim, uma melhor compreensão dessa complexa interação pode contribuir para o desenvolvimento de uma nova abordagem para o controle desse parasito. Este estudo avaliou a comunidade bacteriana em ovinos Corriedale, naturalmente infectados com *H. contortus*, classificados em baixas cargas parasitárias (LC), intermediárias (IC) ou altas (HC), ao longo de dez meses. Amostras de fezes foram coletadas mensalmente para a contagem de ovos (OPG), e o sequenciamento do gene 16S rRNA foi realizado em cinco animais de cada grupo. O OPG médio foi de 2.635 ± 105 para HC, 845 ± 129 para IC e 110 ± 70 para LC, com uma diferença significativa (p = 0,0001). Firmicutes, Proteobacteria e Spirochaetes foram mais abundantes no grupo HC. 102 gêneros bacterianos apresentaram diferenças significativas entre os grupos LC e HC. A Beta diversidade foi estatisticamente diferente (p<0,005) para HC em comparação com os outros dois grupos. Também encontraram-se comunidades diferentes entre LC e HC. *Sediminispirochaeta, Oribacterium*, *Alloprevotella*, *Prevotellaceae*_UCG-001, *Prevotellaceae*_UCG-003, *Ruminiclostridium*_6 e *Ruminococcus*_1 foram significativamente mais abundantes no grupo LC e IC. Acetobacter e Methanocorpusculum tiveram uma redução significativa no grupo LC. Gêneros bacterianos relacionados à baixa emissão de metano e melhor eficiência alimentar estavam significativamente presentes no grupo LC. Portanto, uma melhor compreensão do papel da interação hospedeiro-parasita pode contribuir para melhorar o controle de *H. contortus*.

## Introduction

Infections caused by the gastrointestinal nematode *Haemonchus contortus* are one of the main reasons for economic losses in sheep breeding (Miller et al., 2012; Zajac, 2006). Attempts to control parasitic infections, especially helminths, have increased the frequency and doses of anthelmintic drugs, leading to parasite resistance to practically all the available active ingredients (Salgado & Santos, 2016). Studies have investigated different strategies to control helminths, including rotational grazing, crop-livestock integration, genetic selection, biological control (fungi and bacteria), herbal medicines, and vaccines (Molento et al., 2013; Sinott et al., 2012), yet more efficacious options are needed to prevent infection.

The bacterial community in the ruminant gastrointestinal tract (GIT) involves several biological functions, such as digestion, cellulose degradation, and modulation of the immune response (Arshad et al., 2021; Hooper et al., 2002). Evidence suggests that the onset of microbial colonization occurs already in the fetal period; however, after birth, a major change in the composition is observed in approximately the first 60 days of life (Yin et al., 2021). In this period, the bacteria bacterial community is primarily constituted by Actinobacteria, Firmicutes, and Proteobacteria phyla (Bi et al., 2021). Lately, there have been reports of the interaction of parasites and bacterial communities in the GIT, with some interaction affecting parasite infection (Mamun et al., 2020; Watkins et al., 2021).

There is a growing interest in understanding the relationship between resistant and susceptible sheep phenotypes to gastrointestinal nematodes and their bacterial community (Cortés et al., 2020; Castilla Gómez de Agüero et al., 2022; Mamun et al., 2020; Niciura et al., 2024; Paz et al., 2022; Watkins et al., 2021). Sheep with different parasitic genera and loads show significant differences in the bacterial community present in GIT (Paz et al., 2022; Cortés et al. 2020)*.*

Better understanding the role of host bacterial community-parasite interaction in parasite susceptibility could contribute to the development of novel parasite control options (Cortés et al., 2020; Castilla Gómez de Agüero et al., 2022; Mamun et al., 2020; Niciura et al., 2024; Paz et al., 2022; Watkins et al., 2021). Niciura et al. (2024) recently reported that parasitic genes, biological pathways, and microbiomes play an important role in *H. contortus* resistance in sheep flocks, emphasizing the complex interaction between breed, parasite, and microbiome.

Therefore, the present study aimed to characterize the bacterial microbiome relationship with the level of *Haemonchus contortus* eggs shed in feces in naturally infected sheep.

## Material and Methods

### Evaluation of the parasitic load

A producer from the municipality of Pedro Osório, RS, Brazil, South of Brazil (31º51'51” S-52º49'24” W) kindly provided animals. Forty Corriedale sheep (18 to 20 months old) of both sexes (25 males and 15 females) randomly selected from a herd of about 150 individuals were monitored for their parasitic load over a span of ten months. The sheep were kept in a native field and did not receive antimicrobials or antihelminth during the study period. The FAMACHA score was used to monitor anemia in the experimental animals. Fecal sample collections were performed monthly to count eggs per gram of feces (EPG). Eggs were measured in fecal samples (~30g) collected directly from the rectum and quantified following the modified Gordon and Whitlock technique. The Gordon & Whitlock (1939) technique uses a flotation solution to separate eggs from feces. After filtration, the eggs are counted under a microscope, and the results are reported as the number of eggs per gram of feces. One gram of each sample was immediately frozen at −70°C for subsequent sequencing. To categorize each animal, we relied on the study by Hupp et al. (2018), which suggests parasite load levels for intervention with anthelmintics. Accordingly, the animals were divided into Low count (LC) up to 200 EPG, Intermediary count (IC) between 200 and 1000 EPG, and High count (HC) higher than 1000 EPG. EPG results were submitted to the Two-Way ANOVA followed by Tukey’s post-hoc test in GraphPad Prism 9 software to assess statistical differences between groups. Following the results, the animals were grouped into LC for counts to 200 EPG, IC for animals that presented between 300 - 1000, and HC for sheep above 1000 EPG. Nematodes were identified using coproculture technique for all collections (Roberts & O’Sullivan, 1950).

### Bacterial community profile analysis

From the forty examined sheep for egg count, five animals (three males and two females, 18 to 20 months old) grouped in LC, four in IC, and five in HC were selected for amplification and sequencing of amplicons from feces samples. After grouping the animals into different groups, fecal samples from day 0 and day 90 were selected for gene sequencing. For this bacterial community analysis, DNA was extracted using the QIAamp PowerFecal Pro DNA Kit, and the V3-V4 region of the 16S rRNA gene was amplified using primers Bakt_341F (5’- CCTACGGGNGGCWGCAG-3’ (Wang & Qian, 2009) and Bakt_806R (5’- GGACTACHVGGGTWTCTAAT -3’(Caporaso et al., 2012). The obtained amplicons were sequenced in *paired-end* (2X300 bp) with Miseq Reagent Kit V3 R (600 cycles) on the MiSeq Sequencing System platform (Illumina, Brazil).

The processing and analysis of sequencing data were conducted in R language (R v. 3.6.0) (R Core Team, 2024). The raw readings were filtered in dada2 package (Callahan et al., 2016) with the parameters trimLeft = c (18, 23), truncLen= c (295, 200), maxN=0, maxEE=6 and truncQ=2. The parametric error model was used from the filtered data to learn the error rate of the total set of sequences. The Amplicon Sequence Variant (ASV) table was created using the “makeSequencetable” function, and the chimeric sequences were removed by dada2 using the “removeBimeraDenovo” command. Using the Silva SSU 138 database (Quast et al., 2013), the taxonomy was assigned in the DECIPHER package (Wright, 2016), defining the classification in 6 taxonomic levels. Later, a phyloseq object was created for data manipulation (McMurdie & Holmes, 2013). Normalization of the ASV table was performed by centered log-ratio (clr) in the microbiome package (Lahti & Shetty, 2018), using the “transform” function.

The alpha diversity indices (Shannon and Simpson) for each sample were calculated using the VEGAN package (Dixon, 2003). The group values were compared using the Mann-Whitney test to find significant differences between individual samples. To calculate beta diversity, the Aitchison matrix was chosen using PCA (Principal Component Analysis), and PERMANOVA was calculated using the “adonis” function, setting the number of permutations to 99. The Wilcoxon non-parametric test, followed by Bonferroni correction, assessed the taxonomy ranks exhibiting statistically significant differences among the LC, IC, and HC groups. The ggpubr package (Kassambara, 2020) was used with the “stat_compare_means” function. Graphic visualization was performed using the ggplot2 package (Villanueva & Chen, 2019).

## Results

### Evaluation of the parasitic load

The mean values of ten months of EPG in the evaluated groups were 2.635± 105 for the animals with the highest counts (HC), 845±129 for an intermediary (IC), and 110± 70 for the group with the lowest counts (LC). Two-way ANOVA followed by Tukey’s post-hoc test showed statistical significance between the three experimental groups (p < 0.0001) (Table 1). *Haemonchus contortus* was the predominant nematode during the experiment, representing more than 90% of the parasites, followed by *Teladorsagia circumcincta* and *Trichostrongylus columbriformis*.

**Table 1:** Fecal egg counts per gram (EPG). The data represents the mean +- standard deviation (STD) of the mean of ten months of EPG for the Low (LC), Intermediary (IC) and High count (HC) animals. The Two-Way ANOVA followed by Tukey’s post-hoc test indicates that all groups differ statistically.

**Collect**	**LC**		**IC**		**HC**		
	**Mean**	**STD**	**Mean**	**STD**	**Mean**	**STD**	**p-value**
1°	0	0	600	260	2900	595	<0.0001
2°	200	100	750	208	2500	490	<0.0001
3°	100	70	1000	50	2550	585	<0.0001
4°	200	60	1000	200	2600	590	<0.0001
5°	100	50	900	245	2700	400	<0.0001
6°	0	0	800	255	2600	575	<0.0001
7°	100	100	750	240	2650	350	<0.0001
8°	100	0	750	260	2650	575	<0.0001
9°	100	50	1000	243	2650	530	<0.0001
10°	200	50	900	269	2550	570	<0.0001

### Bacterial community profile analysis

A total of 469.793 (42.708 ± 21.8) raw reads from the two sequencing (day zero and three months) samples from the three groups were received (Table 2). 4.029 ASVs were obtained from the 170.347 (17.034 ± 8.5) readings remaining in the final pipeline step. These sequences were resolved in 19 phyla, 27 classes, 49 orders, 94 families, 202 genera, and 38 species after taxonomy assignment.

**Table 2:** Number of reads remaining in each stage of the pipeline.

**Input**	**filtered**	**denoisedF**	**denoisedR**	**merged**	**nonchim**
74645	74233	54808	57629	28358	26900
74261	73832	55049	56552	27874	26235
59169	58837	46227	49146	24235	23117
64362	63989	50025	52129	26813	25624
36479	30713	22000	25764	13992	11525
13052	11015	7558	8838	5011	4720
21898	18612	13277	15496	8108	7882
37990	32348	22750	27324	14613	13583
32862	27897	22058	25429	11836	11296
32008	27588	21099	24081	13395	13163
23067	19340	14752	17726	6445	6302

Simpson and Shannon indices were calculated to assess the alpha diversity of the samples. The average values found for Simpson's index were 0.998 (±0.0001) and for Shannon's (6.578±0.089). The Mann-Whitney test did not determine a significant difference between groups.

**Figure 1:**
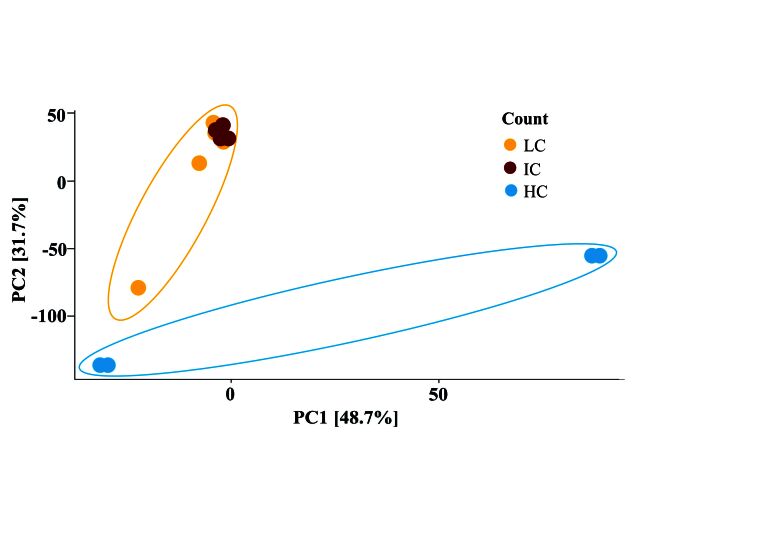
Principal Components Analysis (PCA). The data represents PCA calculated from the Aitchison distance matrix, and demonstrates a distinction in the communities of the LC and HC groups (R = 0.25, p = 0.005).

The comparative analysis between the LC, IC, and HC groups, conducted using PERMANOVA and based on the Aitchison distance matrix (Figure 1), yielded a significant result (R^2^ = 0.25, p < 0.005). Although statistically significant, the R^2^ value suggests that the variation explained by the differences between the groups is relatively low. This indicates that, despite significant variations in beta diversity between the low and high EPG groups, other factors may influence the composition and structure of the communities. Therefore, while the EPG variable impacts community structure, its effect is limited.

**Figure 2:**
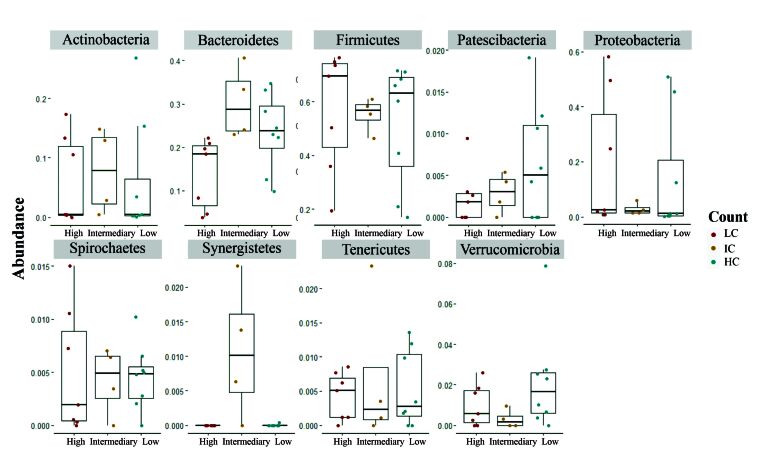
Bacterial phyla between LC, IC, and HC groups. Graphs represent phyla with statistical significance (p < 0.001) inferred using the Wilcoxon test followed by Bonferroni correction. "*" means statistical difference between groups.

When comparing the phyla between the groups using the Wilcoxon test followed by the Bonferroni correction, a total of 9 bacterial phyla were found to be statistically significant among the EPG count groups LC, IC, and HC (p < 0.001) (Figure 2). These results highlight substantial differences in the relative abundance of bacterial phyla, underscoring the impact of EPG counts on the bacterial structure of the communities.

The phyla Firmicutes, Proteobacteria, and Spirochaetes are increased in HC compared to the IC and LC groups. Firmicutes and Proteobacteria are reduced in IC compared to both groups, and Spirochaetes are reduced in LC. There is an increase in Actinobacteria, Bacteroidetes, and Synergistetes in IC, where Actinobacteria is increased in HC and Bacteroidetes are reduced in HC. The phylum Synergistetes shows no statistical difference between the HC and LC groups. Patescibacteria, Tenericutes, and Verrucomicrobia appear to increase in LC, with Patescibacteria and Tenericutes reduced in HC compared to IC and LC and Verrucomicrobia reduced in IC compared to the other groups. At the Class level, 18 taxa showed statistical differences between the LC and IC groups (Figure S1). Between IC and HC, 17 classes differed (Figure S2), while 15 taxa are significantly different between LC and HC (Figure S3).

**Figure 3:**
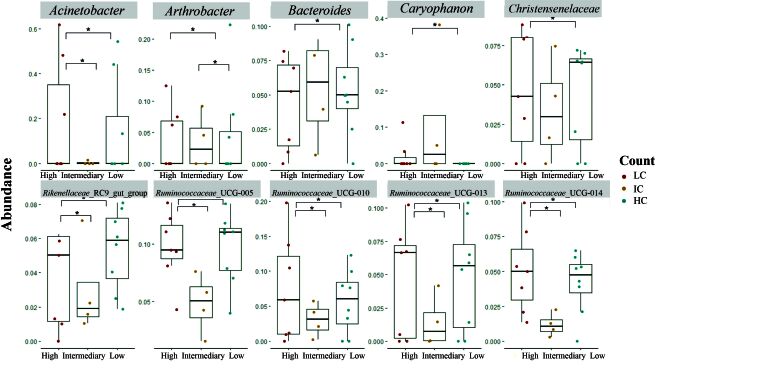
Bacterial abundance at genus level with statistical significance between LC, IC, and HC groups (p < 0.05) inferred using the Wilcoxon test followed by Bonferroni correction. "*" means statistical difference between groups.

The 102 taxa were found to have statistical differences between the LC and HC groups at the genus level (Figure 3 and Table S1). Between the LC and IC groups, 14 genera demonstrated statistical significance, 12 of which were reduced in LC (*Acidovorax, Acinetobacter, Advenella, Anaeroplasma, Camamonas, Desulfomicrobium, Fonticella*, *Macellibacteroides*, *Petrimonas*, *Proteiniphilum, Sphingomonas* and *Syntrophomonas*) and *Arthrobacter* and *Ureaplasma* increased (Table S2). 18 genera were observed to be different between the HC and IC (Table S3), with *Acinetobacter*, *Akkermansia*, *Alistipes*, *Bacteroides*, *Christensenellaceae*_R-7_group, *Macellibacteroides, Ruminicoccaceae_*UCG_014*, Solibacillus* and *Treponema*_2 increased in HC and *Fonticella*, *Lachnospiraceae*_NK4A136, *Petrimonas*, *Proteiniphilum*, *Ruminicoccaceae*_UCG_005, *Ruminicoccaceae*_UCG_010, *Ruminicoccaceae*_UCG_013, *Rikenellaceae*_RC9_gut_group and *Syntrophomonas* in IC.

## Discussion and Conclusion

This study compared and characterized the gastrointestinal (GIT) bacterial community in sheep in natural grass and naturally infected with *H. contortus*. Based on ten months of EPG evaluation, sheep groups were separated into High, Intermediate, and Low parasite loads. Then, the bacterial community of the three characterized groups was evaluated.

The bacterial community was characterized into 19 phyla, 27 classes, 49 orders, 94 families, 202 genera, and 38 species after taxonomy assignment. Evaluating the alpha diversity indices, we did not find differences among the Low Count (LC), Intermediary Count (IC), and High Count (HC) groups, in agreement with a previous study (Paz et al., 2022). Beta diversity demonstrated two distinct microbial communities in LC and HC, whereas the IC group did not show differences compared to the LC and HC groups. It is worth considering that the IC group had ~8 fold more EPG than the LC group, whereas the HC had ~28-fold higher EPG than the LC group.

Paz et al. (2022) reported the different bacterial communities in sheep infected with *T. circumcincta* and *T. colubriformis*. A high parasite burden was associated with a change in bacterial community composition, principally differentiating the relative abundances of Firmicutes and Bacteroidetes, taxa involved in the digestion of fibers and complex polysaccharides, and the production of short-chain fatty acids. We also observed similar results with a more robust analysis pipeline in the present study.

Curiously, Paz et al. (2022) did not find Actinobacteria, Chloroflexi, Elusimicrobia, Synergistetes, and Tenericutes phyla in the final portion of GIT, which were found in our study. The same occurred for *Coprococcus*, *Turicibacter*, *Mycoplasma*, *Ureoplasma*, *Olsenella*, *Clostridium*, *Lachnosclostridium*, *Flexilinea*, *Blautia* and *Elusimicrobium* genera. *Eubacterium*, *Oscillibacter,* and *Ruminococcus* were the most abundant genera found by Paz et al. (2022), while in our observations, *Ruminococcaceae* were dominant, followed by *Christensenellaceae* and *Bacteroides*. One may consider the differences between studies to be the parasite (*H. contortus* versus *T. circumcincta* and *T. colubriformis*); however, this could not exclude other aspects such as sheep breed, grass, year season, etc. Nonetheless, these points were out of the present study's scope.

Castilla Gómez de Agüero et al. (2022) studied microbial differences communities between susceptible and resistant Navajo-Churro breed infected with *T. circumcincta* but found no significant difference between the two groups. Infection caused by *T. circumcincta* can increase the abundance of pro-inflammatory microorganisms, such as *Sutterella, Prevotella,* and *Porphyromonas*, genera that can contribute to the parasite pathogenesis, as they promote inflammation of the abomasal mucosa (Cortés et al., 2020).

Different studies have shown that breed can have a significant influence on the bacterial community (Cholewińska et al., 2020; Castilla Gómez de Agüero et al., 2022; Mamun et al., 2020; Niciura et al., 2024; Paz et al., 2022; Watkins et al., 2021). Animals of the “old-type Polish Merino” breed, for example, have Bacteroidetes as the dominant phyla, while Firmicutes is the most representative in Merino and Navajo-Churro breeds (Cholewińska et al., 2020; Castilla Gómez de Agüero et al., 2022; Mamun et al., 2020). Our findings agree with those authors; we also observed that Firmicutes phyla are the most representative phylum in the Corriedale breed. However, from our data, we can only speculate that the breed plays a role since other factors can play a role in establishing phyla in animal TGI.

Mamun et al. (2020), studying the stability of bacterial community in Merino sheep, reported an increase in Firmicutes. In this study, we observed a reduction of Firmicutes in the LC group. However, our findings agree in different differentially abundant taxa such as Actinobacteria, Spirochaetes, and Proteobacteria, which decreased in the lowest shedding animals in both studies. Our results indicate a reduction of Proteobacteria in the animals with low loads of *H. contortus*. Proteobacteria is often a phylum considered harmful to the hosts, as it is composed of Gram-negative bacteria capable of causing dysbiosis, such as *Escherichia coli* and *Campylobacter* spp*.* (Mamun et al., 2020).

Niciura et al. (2024), similar to the results obtained in our study, observed that infection with *Haemonchus contortus* affects the composition and diversity of the microbiota in parasitized sheep. Their study indicates that such an infection can lead to dysbiosis in microbial communities, resulting in changes to their composition. Additionally, the presence of *H. contortus* induces alterations in the abomasum and rumen, impacting protein digestion capacity and amino acid allocation (Niciura et al., 2024). It is important to highlight that both studies contribute distinct insights into the interaction between the parasite, host, and microbiota. The study by Niciura et al. (2024), in particular, conducted experimental infections of *H. contortus* in Nova Morada sheep, offering significant insights into the relationship between the microbiota and this parasite.

Our findings demonstrated that 102 genera are statistically significant between the LC and HC groups. For genera positively related to methane emission, such as *Acetobacter* (Cunha et al., 2019; Tanca et al., 2017), a significant reduction in the LC group was found. In line with our results, animals experimentally infected with *H. contortus* and *T. colubriformis* increased enteric methane emissions due to an increase in methanogenic microorganisms, which can result in a more significant loss of energy ingested by the animal (Corrêa et al., 2021). In our results, 14 genera differ between the LC and IC and 18 between HC and IC. The genus *Fonticella* is increased in IC, and some species are related to the fermentation of glucose and other sugars into acetate and ethanol (Fraj et al., 2013). The same occurs with *Proteiniphilum*, considered one of the most abundant genera in animals fed crude protein, which decreased the concentration of enteric methane and related to milk's protein content (Kim et al., 2020).

*Sediminispirochaeta* and *Oribacterium* are bacterial genera found in animals with good feed efficiency. They are involved in the degradation of the cell wall and assist in the digestibility of nutrients (Marie-Etancelin et al., 2021). Both were found to be more abundant in the LC group, suggesting that animals with low load of parasites may also have good feed efficiency. Fibrolytic bacteria such as *Prevotellaceae*_UCG-001, *Prevotellaceae*_UCG-003, *Ruminiclostridium*_6, and *Ruminococcus*_1 were also increased in the LC group. This group of bacteria is responsible for the degradation of vegetal fibers, which play a fundamental role in the feeding and nutrition of ruminants (Koike & Kobayashi, 2009). Notably, there is also an increase in low count sheep for the genus *Alloprevotella*, which is positively related to fat deposition in sheep (Cheng et al., 2022).

Based on our findings, it was possible to identify a distinct bacterial community in Corriedale sheep naturally infected by *H. contortus* based on their level of parasite shedding. Our findings suggest that microbial composition is related to the degree of infection of sheep under natural field infection conditions. Our study has limitations, such as the small number of sequenced samples and the selected collection days. Nevertheless, we believe our findings are significant for understanding the role of bacterial communities in the parasite load of naturally infected animals. The changes in bacteria between groups could allow the development of tools needed for hemoncose control.

## Supplementary Material

Supplementary material accompanies this paper.

Figure S1. Bacterial abundance at Class level with statistical significance between LC and IC groups.

Figure S2. Bacterial abundance at Class level with statistical significance between IC and HC groups.

Figure S3. Bacterial abundance at Class level with statistical significance between LC and HC groups.

Table S1. Egg count per grams of feces over a 10-month period.

Table S2. Bacterial abundance at genus level with statistical significance between LC, IC, and HC groups.

Table S3. Bacterial abundance at genus level with statistical significance between LC and IC groups.

This material is available as part of the online article from https://doi.org/10.1590/S1984-29612025005
