# Supplementary material for: Fecal shedding level of Haemonchus contortus is associated with gastrointestinal bacterial microbial composition in naturally infected sheep
Source: Rev Bras Parasitol Vet. 2025 Feb 3;34(1):e017724. doi: 10.1590/S1984-29612025005 (PMC11801252; doi:10.1590/S1984-29612025005)
Supplement: Figure S1. [file rbpv-34-1-e017724-suppl01.pdf]

Supplementary material

Figure S1. Bacterial abundance at Class level with statistical significance between LC and IC groups.

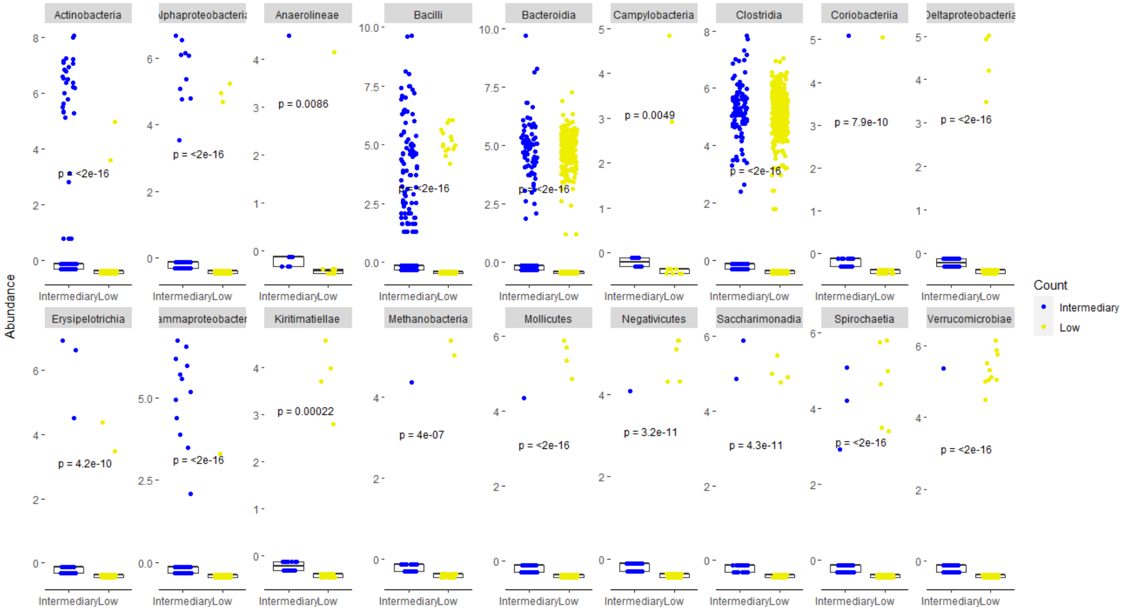

Figure S2. Bacterial abundance at Class level with statistical significance between IC and HC groups.

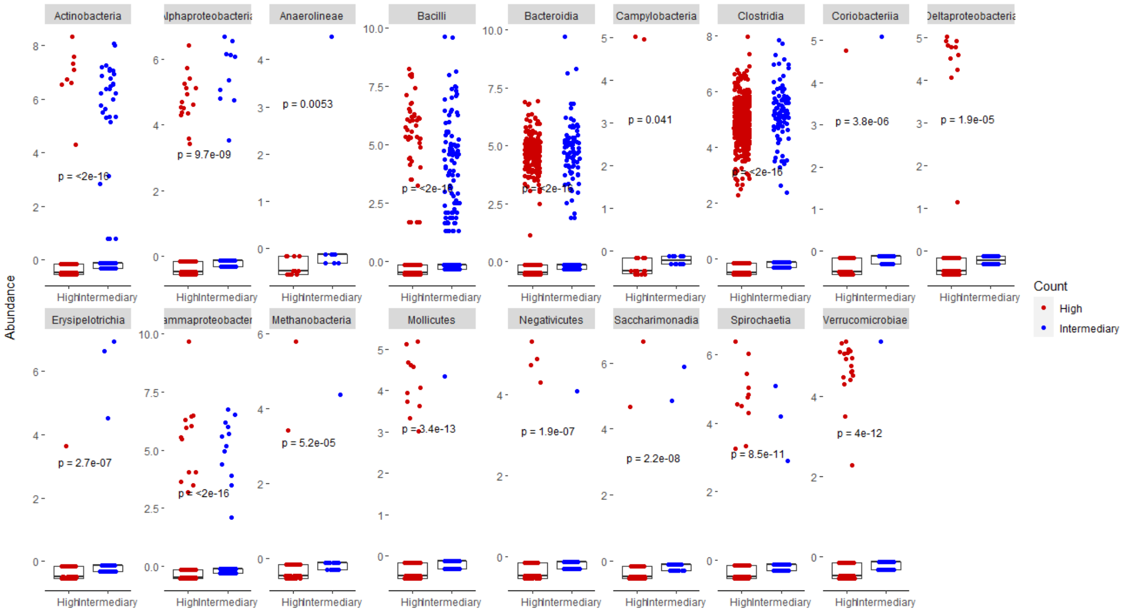

Figure S3. Bacterial abundance at Class level with statistical significance between LC and HC groups.

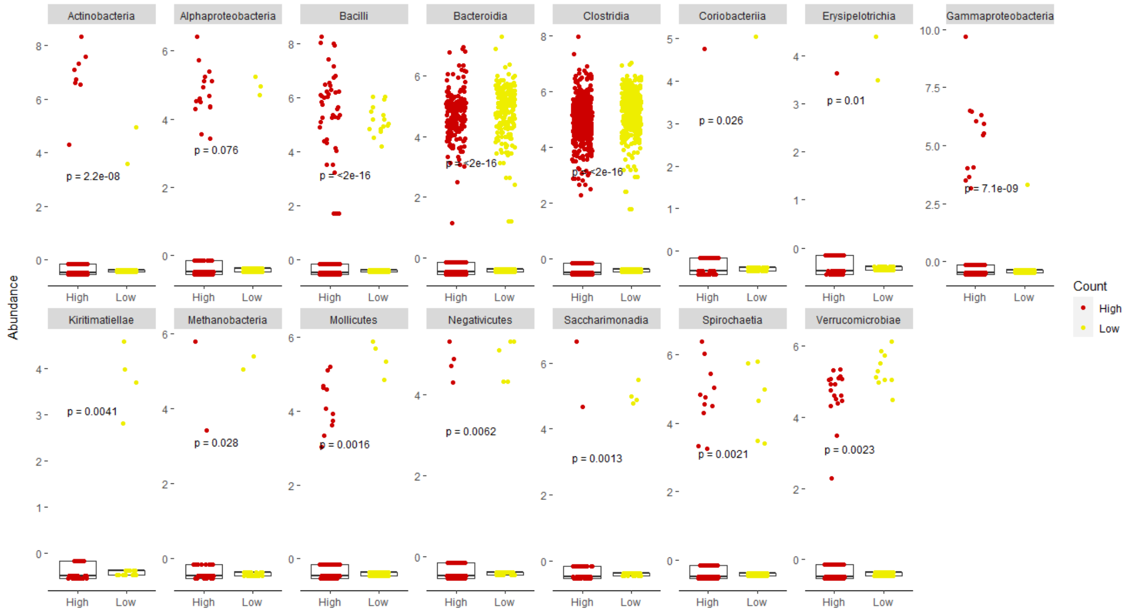

Table S1. Bacterial abundance at genus level with statistical significance between LC and HC groups.

Gênero	LC	HC	P-value
Acetobacter	-	+	0.032
Acinetobacter	-	+	1.1e-08
Agathobacter	+	-	0.002
Akkermansia	+	-	6e-11
Alistipes	+	-	1.7e-12
Alloprevotella	+	-	0.0036
Allorhizobium-Neorhizobium-Pararhizobium-Rhizobium	+	-	0.016
Anaerocella	+	-	0.077
Anaeroplasma	-	+	0.0087
Anaerovorax	+	-	0.00097
Arthrobacter	+	-	4.4e-06
Atopostipes	+	-	0.04
Bacteroides	-	+	2e-16
Butyricicoccus	-	+	0.016
Butyrivibrio	-	+	0.019
Caldicoprobacter	-	+	0.016
Campylobacter	+	-	0.045
Candidatus Saccharymonas	+	-	0.0035
Candidatus Soleaferrea	-	+	0.0014
Candidatus Stoquefichus	-	+	0.016
Caproiciproducens	+	-	0.032
Caryophanon	-	+	0.0049
Cerasicoccus	-	+	0.011
Christensenellaceae_R-7_group	+	-	2.5e-05
Clostridium_sensu_stricto_1	+	-	0.029
Coprococcus_2	+	-	0.015
Corynebacterium_1	+	-	1e-12
dgA-11_gut_group	-	+	1.7e-06
Elusimicrobium	-	+	0.0092
Enterococcus	+	-	0.011
Escherichia/Shigella	-	+	0.032
Family_XIII_UCG-001	+	-	0.032
Fermentimonas	+	-	0.04
Fibrobacter	-	+	0.00046
Flavonifractor	-	+	5 e-04
Flexilinea	+	-	0.016
GCA-900066225	+	-	0. 025

Helcococcus	-	+	0.032
Herbinix	+	-	0. 0062
Lachnoclostridium	-	+	0.041
Lachnospiraceae_FCS020_group	-	+	0.046
Lachnospiraceae_NK4A136_group	-	+	0.0024
Lachnospiraceae_XPPB1014_group	+	-	0. 055
Lactobacillus	+	-	0. 042
Lochnoclostridium_10	+	-	0. 0033
Lysinibacillus	-	+	0. 0028
Macellibacteroides	+	-	0. 0019
Maihella	-	+	0.036
Methanobrevibacter	+	-	0.037
Methanocorpusculum	-	+	0.016
Mucispirilum	-	+	0.016
Mycoplasma	-	+	0.032
Olsenella	+	-	1e-12
Oribacterium	-	+	0.016
Oscillibacter	-	+	0. 0023
Paenibacillus	-	+	0. 0019
Pedobacter	+	-	0. 00034
Petrimonas	+	-	0. 0011
Phascolarctobacterium	-	+	0.0022
Porphyromonas	+	-	0. 0083
Prevotella_1	+	-	3.6e-05
Prevotellaceae_Ga6A1_group	+	-	0. 016
Prevotellaceae_UCG-001	+	-	4.9e-06
Prevotellaceae_UCG-003	+	-	3.7e-08
Prevotellaceae_UCG-004	-	+	0.011
Proteiniphilum	+	-	5.8e -05
Pseudobutyrivibrio	+	-	0.016
Psychrobacillus	-	+	8.2e -05
Pyramidobacter	+	-	0.032
Rhodococcus	+	-	1e-12
Rikenellaceae_RC9_gut_group	+	-	2.2e -16
Roseburia	+	-	0. 034
Ruminiclostridium	+	-	0. 0076
Ruminiclostridium_1	+	-	0. 0016
Ruminiclostridium_5	+	-	0. 0058
Ruminiclostridium_6	+	-	0.0042
Ruminococcaceae UCG 010	-	+	0.007

Ruminococcaceae UCG 013	-	+	0.027
Ruminococcaceae UCG 014	+	-	0.00018
Ruminococcaceae_NK4A214_group	+	-	7.8e -12
Ruminococcaceae_UCG-002_group	+	-	9e -07
Ruminococcaceae_UCG-004_group	+	-	0. 0092
Ruminococcaceae_UCG-005_group	+	-	2.2e -16
Ruminococcaceae_UCG-009_group	-	+	6.2e -05
Ruminococcaceae_UCG-010_group	+	-	2.2e -16
Ruminococcaceae_UCG-011_group	+	-	0. 025
Ruminococcaceae_UCG-013_group	-	+	2.2e -16
Ruminococcaceae_UCG-014_group	-	+	2.2e -16
Ruminococcus_1	+	-	0.039
Ruminococcus_2	-	+	0. 00041
Ruminofilibacter	+	-	0. 021
Saccharofermentans	+	-	0. 00067
Sanguibacteroides	-	+	0.016
Sediminispirochaeta	+	-	0.016
Solibacillus	-	+	1.2e -12
Sphingomonas	+	-	0. 052
Streptococcus	+	-	0.016
Succiniclasticum	+	-	0.02
Syntrophomonas	+	-	0.04
Treponema_2	+	-	7.7e -08
Tyzzerella	+	-	0. 016

Table S2. Bacterial abundance at genus level with statistical significance between LC and IC groups.

Genera	LC	IC	P-value
Acidovorax	-	+	8e-05
Acinetobacter	-	+	2e-18
Advenella	-	+	0.008
Anaeroplasma	-	+	8.8e-07
Arthrobacter	+	-	0.019
Camamonas	-	+	7.2e-05
Desulfomicrobium	-	+	8.9e-08
Fonticella	-	+	0.0049
Macellibacteroides	-	+	7.9e-08
Petrimonas	-	+	0.00029
Proteiniphilum	-	+	1.8e-07
Sphingomonas	-	+	6e-07
Syntrophomonas	-	+	0.0052
Ureaplasma	+	-	0.00045

Table S3. Bacterial abundance at genus level with statistical significance between IC and HC groups.

Gênero	HC	IC	P-value
Acinetobacter	+	-	9.3e-08
Akkermansia	+	-	0.0088
Alistipes	+	-	0.00023
Bacteroides	+	-	1.2e-07
Christensenellaceae_R-7_group	+	-	0.0012
Fonticella	-	+	0.012
Macellibacteroides	+	-	0.0089
Lachnospiraceae_NK4A136	-	+	1.2e-06
Petrimonas	-	+	0.00069
Proteiniphilum	-	+	2.6e-06
Ruminicoccaceae_UCG_005	-	+	0.001
Ruminicoccaceae_UCG_010	-	+	0.001
Ruminicoccaceae_UCG_013	-	+	0.001
Ruminicoccaceae_UCG_014	+	-	0.001
Rikenellaceae_RC9_gut_group	-	+	9.9e-11
Solibacillus	+	-	0.013
Syntrophomonas	-	+	0.012
Treponema_2	+	-	0.0058
